# Maternal Bonding in Early Infancy Predicts Childrens' Social Competences in Preschool Age

**DOI:** 10.3389/fpsyt.2021.687535

**Published:** 2021-08-19

**Authors:** Jens Joas, Eva Möhler

**Affiliations:** Department of Child and Adolescent Psychiatry, Faculty of Medicine, Saarland University, Homburg, Germany

**Keywords:** bonding, social competences, social skills, child development, longitudinal study

## Abstract

**Background:** There are many studies on mother-child-bonding with little theoretical doubt that better bonding may have a positive effect on further social development. However, there is hardly any empirical evidence. In particular, there is a lack prospective longitudinal studies.

**Methods:** As part of a longitudinal study, bonding was assessed in a community sample of 97 healthy mothers using the Postpartum Bonding Questionnaire (PBQ) 6 weeks after birth of their child. Social competencies in the offspring were assessed using the Self- and Other-oriented Social Competencies (SOCOMP) at 5.5 years of age. A potential correlation between bonding and social competencies was tested using Spearman Rank Correlation.

**Results:** Retention rate over 5.5 years was 77.23%. Lower Maternal Bonding Impairment Scores 6 weeks postnatally were positively related to childrens' social competences at 5.5 years of age.

**Conclusion:** The present data confirm a positive and long-term influence of bonding on social skills and provide further evidence of the importance of parent child bonding for child development in general. This result should give reason to further investigate this relationship in depth, causally and at later points in time.

## Introduction

Bonding and early social experiences are assumed to be associated with a more healthy social and emotional development, to protect against stress and make children more resilient (1). Bonding is defined in developmental psychology as the emotional connection from parents to their children, in contrast to attachment, which is the emotional connection of the child toward its caregiver. The opportunity for the development of bonding is assumed to have its peak in the 1st min and hours after birth, especially in the close physical contact and the reactions of the helpless infant seeking comfort, protection, warmth and nourishment from the parents' behavior (2–4). This first phase is also postulated to be the “sensitive period” and to equally occur in most mammals (5). Newborns, placed on the mother's abdomen, can instinctively locate and suckle the maternal breast without assistance in their 1st hour of life via their sense of smell/pheromones (6–9).

Undoubtedly, mothers have a large part in establishing social contact with their infant (10), but the newborns are able to interact with her via eye contact, body language and thus in turn elicit linguistic utterances (11). The lack of skin contact with the mother in the first 2 hours after birth alone causes the infant's body temperature to be lower (12). After 1 year, according to Parent-Child-Early Relational Assessment (PCERA) video analyses, the infants tended to be more dysregulated and irritable, their social interactions with mothers were less substantial (also in terms of reciprocal emotional response) (13).

Mothers and infants with 16 h more physical contact shortly after birth showed more reluctance to leave their baby with another person compared to the control group about 4 weeks later during a standardized interview, a medical examination of the baby and a recorded bottle feeding (14). These mothers watched and mostly stood during the examination, tried to calm their infants more, performed more stroking and expressed significantly more eye contact. There have been numerous studies documenting positive and negative effects of a lack of mother-infant (skin) contact in the infant's 1st h (15). Not only was the body and skin temperature lower in children in cots compared to children with mothers, the glucose content in the blood was also lower and they cried and expended significantly more energy (16, 17). Even 20 min of skin contact was associated with a significant reduction in circulating beta-endorphin (18). After 4 postnatal hours, babies with increased physical contact show a majority of longer sleep, a calmer sleep state, more bending and fewer stretching movements (19). Babies who had a stable very low birth weight even showed improved lung function with direct contact in some cases (20).

Early maternal mind-mindedness [“to treat her infant as an individual with a mind rather than merely as a creature with needs that must be satisfied,” (21)] also have been reported to be of influence on the social-cognitive development of her child, affecting the development of empathy skills (22). Social skills in turn, are described to be associated with mental and psychological health (23) as well as—in a negative association—with a wide range of disorders such as anxiety (24), blood pressure (25), substance abuse (26), and problem behaviors such as juvenile delinquency (27). High social skills, on the other hand, have been shown to lead to higher financial and professional success (28). Similar to bonding or also bonding vs. attachment (3) there is a definition and demarcation problem with social competences (e.g., on social skills) (29). According to the literature, studies for attachment and its influence on social competences could be found frequently (30–32), whereas there seems to be a lack of studies regarding bonding and its influence on social skills. It has been described however, that pet bonding of young children, in contrast to simple pet presence, has a positive effect on their social competences and empathy (33). With regard to the methodological problems, the advantages and disadvantages of sociometric or observed recording of social competences in children, we refer e.g., to Foster and Richey (34).

In their review, Alves et al. (35) concluded that a long separation from the mother triggers anxiety and depression—like symptoms in rodents—and is reflected qualitatively and quantitatively in maternal behavior. In a study on foals, separated from their mothers for 1 h, were less socially competent after 1 year, which corresponds to the prepubertal period of these animals. Additionally they were more aggressive and showed withdrawal tendencies (36).

So far however, there has been no study examining the impact of neonatal bonding on childrens' social competences in a prospective longitudinal design. Whether maternal bonding in the neonatal period is related to social competences of children at preschool age in humans is the subject of this study.

## Materials and Methods

### Study Design

In the present study, mothers completed the Postpartum Bonding Questionnaire at 6 weeks after birth. At the age of 5.5 years, the social skills of her child were assessed with a standardized instrument, also based on the mother's assessment.

### Participants

The voluntary sample recruited 2002 and 2003 by Möhler et al. (37) consisted of 101 healthy Caucasian mothers with singleton pregnancies. Inclusion criteria were infant weight over 2,500 g, gestational age > 37 weeks, all APGAR scores > 7 and, generally good health of the baby as evidenced by the first 3 postnatal examinations. The mothers were from urban and rural areas and have been recruited from 4 large local maternity units. Exclusion criteria were an inability to speak and read German, an acute psychiatric disorder of the mother, as well as the use of drugs or medication that pose a risk to the fetus, excessive smoking (more than 5 cigarettes/day) and alcohol consumption during pregnancy.

All participants read the participant information sheet and had the opportunity to ask questions. An informed consent form was read, signed and returned by all child custodians. All participants took part in the study voluntarily and could withdraw their participation at any time without giving a reason.

Of the original 101 mothers, 97 filled out the postpartum Bonding Questionnaire 6 weeks postnatally, 78 still responded after 5.5 years which corresponds to a response rate of 77.23%. This sample also had to be adjusted for 1 outlier and another case due a high number (more than 1 missing value) of missing values, so that the final sample is 76. If there was exactly 1 missing value in a questionnaire, the rounded individual subscale mean of the respective test has been used for this. This was necessary in 9 cases. The flow of participants can be found in Table 1.

**Table 1:** Flow of participants.

	***N***	**%**
N T0	101	100
n T1	97	96.04
Responded and take part T2	78	77.23
Excluded as an outlier	1	0.99
Excluded due to too many missing values	1	0.99
Sample n T2	76	75.25

*T0, prenatal; T1, 6 weeks after birth; T2, 5.5 years after birth*.

### Measures

The Postpartum Bonding Questionnaire (PBQ) (38) measures disturbances in the mother-child relationship based on self-report by the mother using a six-point Likert scale. The questionnaire, consisting of 25 items and 4 subscales (impaired bonding [12 items], rejection and anger [7 items], anxiety about care of the baby [4 items], risk of child abuse [2 items]) and a total score, has satisfactory interrater reliabilities (*Pearson's r* 0.95, 0.95, 0.93, 0.77), except for the 'risk of child abuse' scale. Similar in terms of sensitivity (0.93, 0.57, 0.43, 0.18, in severe cases sensitivity 1.0, 0.89, 0.56, 0.28). In its validation study (39), these values could be approximately replicated (0.82, 0.68, 0.61, 0.13, in severe cases 0.93, 0.88, 0.64, 0.2).

The Social Competences Inventory SOCOMP (40) captures self- and other-oriented social skills in both its parent/teacher version, which was used because of its low threshold, multidimensionality and suitability for the young children in this study, and its child version. Its 25 items, assignable to the 3 main dimensions of self-oriented social skills, other-oriented social skills and positive peer relationships, are based on already established instruments such as the “Strenghts and Difficulties Questionaire” (scales of prosocial behavior and problems with peers) (SDQ) (41) or “The Social Skills Rating System” (SSRS) (42). Self-oriented competencies, defined as achieving one's own goals and satisfying needs in social interactions, are assessed via 10 items, which can be further divided into the subscales leadership, setting limits and social participation. Other-oriented competences, defined there as the extent to which gratifications and the goals of others are taken into account at the same time, are also surveyed by 10 items and offer the subscales prosocial behavior and cooperative behavior. The remaining 5 items of the positive peer relationships scale measure peer relationship quality. All items are rated using a three-point Likert scale. The internal consistency of the items of the parent version is medium to high (43).

### Statistical Analysis

The data were analyzed with IBM SPSS Statistics, version 26. Due to the non-normal distribution of the data (K.-S.-Test: PBQ *p* < 0.001; SOCOMP *p* = 0.02), a Spearman Rank Correlation was calculated. A significance level of 0.05 was used for all statistical tests.

## Results

### Sample

Characteristics of the sample are presented in Table 2. The mothers were aged between 19 and 45 years (*M* = 33.58 years, *SD* = 4.07) at the first time of measurement, non-smokers, did not drink more than an occasional glass of wine or beer during pregnancy and had a term birth. The birth weight of the children ranged from 2,520 g to 4,500 g (*M* = 3486.32, *SD* = 421.15). 57.9% of the children were male, 42.1% female. All mothers were in a stable partnership with the child's father. 19.74% of them had a secondary school leaving certificate, 19.74% had a high school leaving certificate, 60.53% had a university degree and were thus educated above average.

**Table 2:** Characteristics of sample (*n* = 76).

**Characteristic**	***M***	***SD***	**Min**.	**Max**.
Age of mothers in years T1	33.58	4.07	19	45
Birth weight child in grams	3486.32	421.15	2,520	4,500
Newborn gender	n	%		
Male	44	57.9		
Female	32	42.1		
School graduation (mother)	n	%		
Secondary school	15	19.74		
High school	15	19.74		
University	46	60.53		

*T1, 6 weeks after birth*.

### Descriptive Analysis

**Figure 1:**
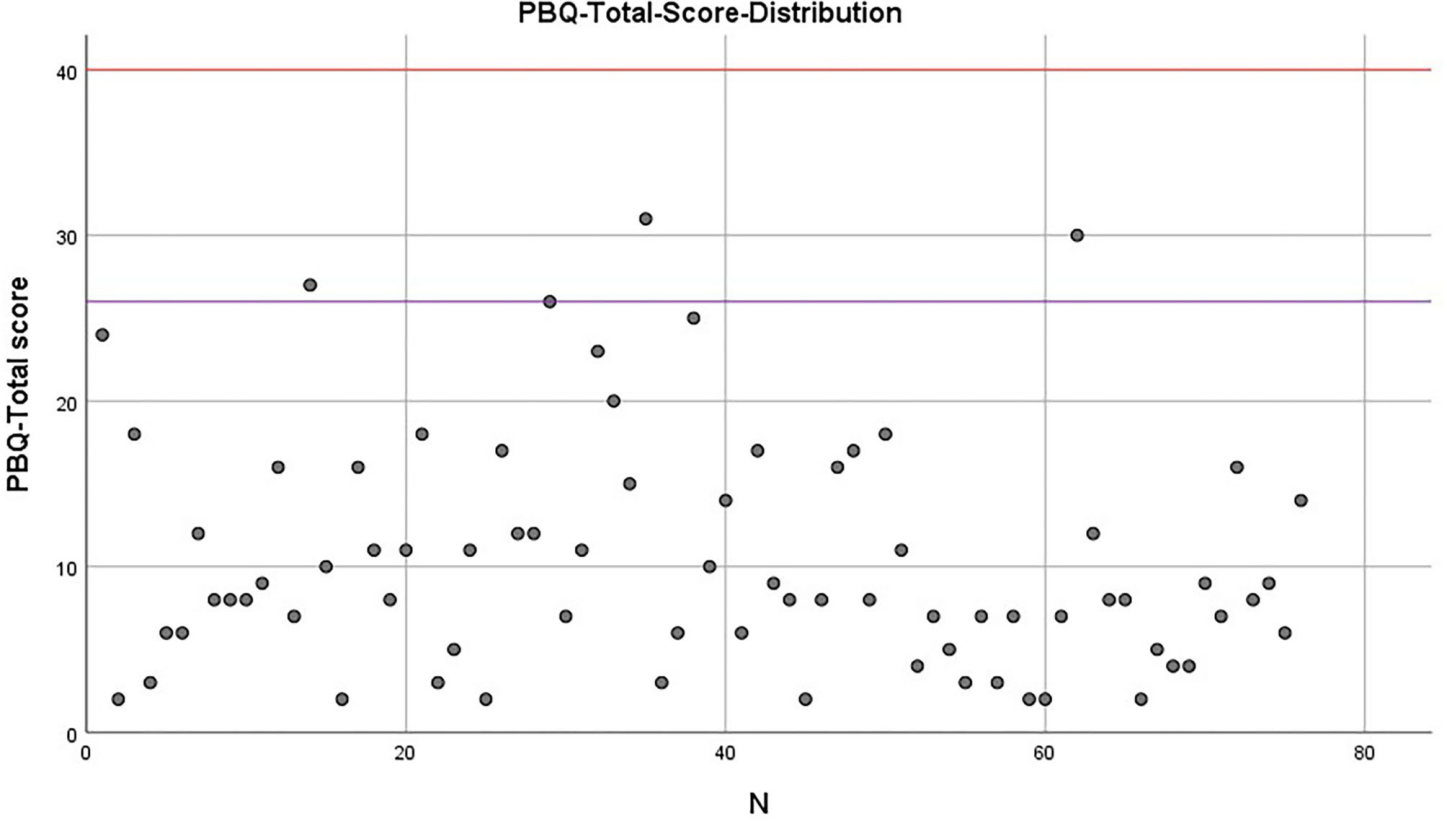
PBQ total score distribution: 4 mothers score above the cut-off of 26 (significant bonding disorder) (purple line), no mother scored above the cut-off of 40 for maternal rejection (red line) (*n* = 76).

Figure 1 shows the distribution of the total PBQ scores (*M*= 10.42, *SD* = 7.01) of the final sample. The auxiliary lines show that only 4 test persons meet or exceed the cut-off (purple line) of 26 for identifying some type of bonding disorder and no mother reaches the value of a maternal rejection (red line). Table 3 illustrates that the cut-off was only exceeded in eight cases in the impaired bonding scale.

**Table 3:** Descriptive statistics of PBQ results (*n* = 76).

	**Total**	**Impaired bonding**	**Rejection and anger**	**anxiety about care of the baby**	**risk of child abuse**
Items	25	12	7	4	2
M	10.42	5.86	2.11	2.33	0.13
SD	7.01	4.10	2.21	1.29	0.38
Minimum	2	0	0	0	0
Maximum	31	17	8	6	2
Cut-off	26 resp. 40	12	17	10	3
n > cut-off	4 resp. 0	8	0	0	0

**Figure 2:**
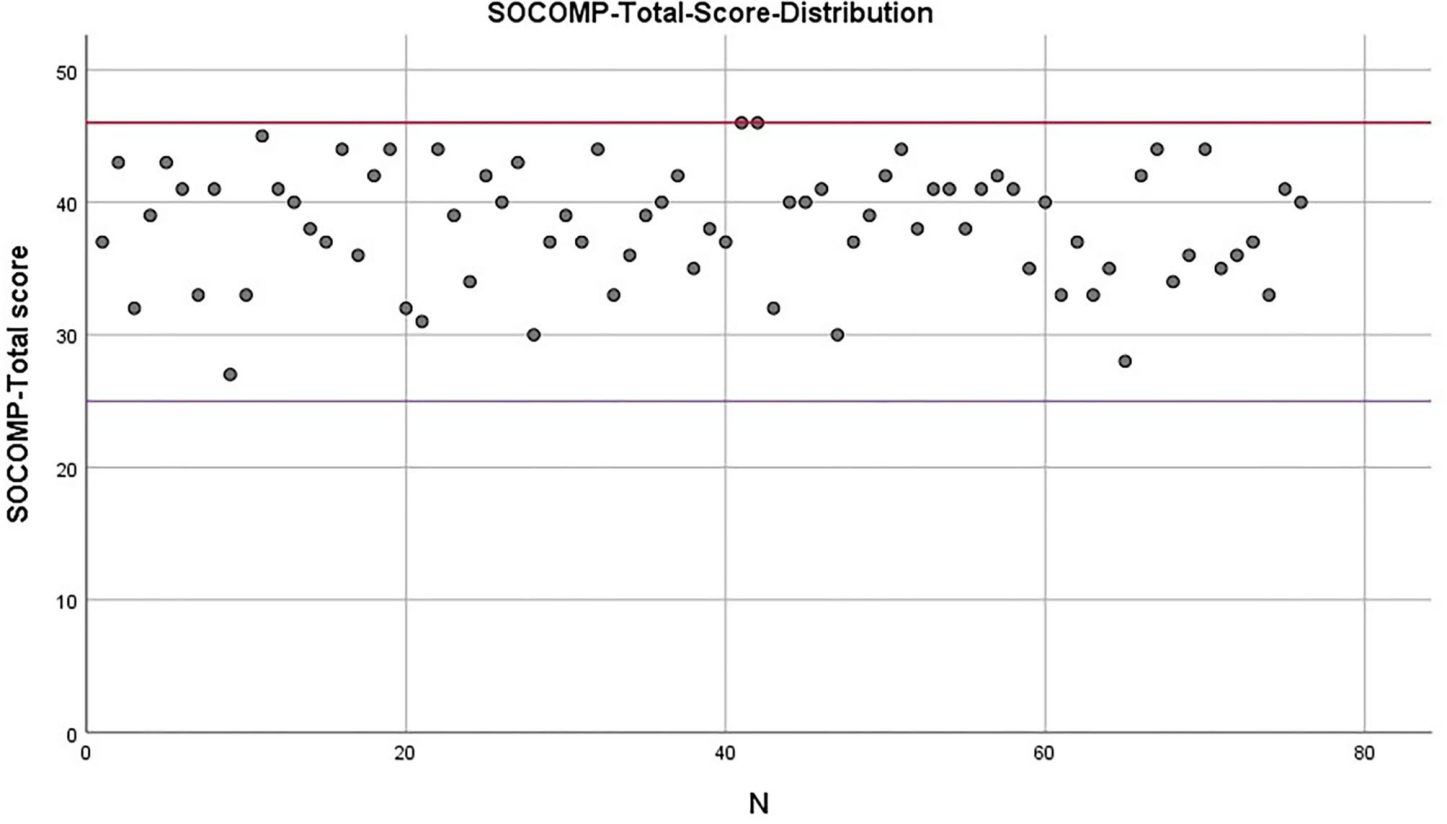
SOCOMP total score distribution with provisional cut-offs equal to 25 (purple line) and lower, standing for low social skills, and 46 (red line) and higher, for high social skills (*n* = 76).

Distribution of data regarding social competences in preschool age can be seen in Figure 2. No child falls below the provisional cut-off value for the total score, the lower limit of which was determined on the basis that each item was agreed to at least proportionally and the upper limit of which reflects the top 10%. Two children lie exactly on the provisional cut-off value (*M* = 38.29, *SD* = 4.41). For the majority of the sample, the social competences are therefore adequate to well-developed. Here, too, it is worth considering the respective subscales (Table 4). While almost a quarter (23.68%) of the children have low self-oriented social competences and no child has conspicuously high values in this area, hardly one child (3.95%) has a low level of other-oriented social competences and 44.74% have high values. The results for peer relationships are even more positive. There, only 1 child has a low, but more than half (53.95 %) have satisfactory social competencies.

**Table 4:** Descriptive statistics of SOCOMP-results (*n* = 76).

	**Total**	**Self-oriented social skills**	**Other-oriented social skills**	**Positive peer relationships**
Items	25	10	10	5
M	38.29	12.47	16.57	9.25
SD	4.41	2.24	2.77	1.10
Minimum	27	8	10	5
Maximum	46	17	20	10
Provisional cut-off	25/46	10/18	10/18	5/10
n < /> provisional cut-off	0/2	19/0	3/34	1/41

The mothers' physical and psychological symptoms, assessed at both time points by the Symptom Checklist 90 revised [SCL-90-R; original (44): german version (45)] showed a remarkably low level (Global severity index after 6 weeks: *M* = 0.21, *SD* = 0.15; after 5.5 years: *M* = 0.17, *SD* = 0.18), with regard to global severity index after 6 weeks only two mothers [T60 and T61], after 5.5 years only one mother [T65] scored above the cut off.

### Correlation of Maternal Bonding With Social Skills

Even in this socially protected and well-adjusted sample, a Spearman Rank Correlation between the PBQ total scores and the SOCOMP total scores revealed a significant correlation (Table 5). High PBQ total scores (high bonding impairment) are therefore negatively related to SOCOMP total scores [*rs*(74) = −0.31, *p* = 0.01]. Less optimal mother-child bonding is shown to predict lower social skills in the child. According to Cohen (46), we are consequently in the range of a moderate correlation (*r* = 0.30).

**Table 5:** Spearman Rank Correlation of bonding impairment assessed 6 weeks after birth with social competencies assessed at 5.5 years.

**Variable**	***n***	***M***	***SD***	**1**	**2**
1. PBQ-Total score	76	10.42	7.01		−0.31^*^
2. SOCOMP-Total score	76	38.29	4.41	−0.31^*^	

* *p < 0.05*.

## Discussion

Our data in a healthy community sample indicate an early influence of maternal bonding on childrens' social skills in preschool age. The mechanisms behind this association are presumed to be interactional, however a genetic contribution to this association cannot be excluded. Unfortunately, there are only a few long-term studies with humans, but most of them show positive effects of early maternal bonding (13, 14). Prospective longitudinal studies with humans are difficult, not only from an ethical point of view, but also in general in this area, as there are many influencing variables (e.g., social events, interactions, also with peers, psychopathology, substance abuse). Our sample tried to control these factors with strict inclusion criteria. Long-term studies with animals underline our data showing the negative aspects of a lack of bonding [e.g., on social behavior (36)], even if they are not easily transferable to humans (47).

Defining social competences is difficult. There are also advantages and disadvantages in methodological recording (34). In our case, they were assessed sociometrically and based on mothers' judgements. Self-assessments, which we could have compared, were not yet available in this age group and observations would only have been possible via excerpts. Of course, social skills are not only influenced by bonding, but also by a variety of other factors. Dodge (48) considers social skills as an interaction between biologically determined abilities and environmental factors. On the side of biological factors temperament (49), temperamental surgency and emotion regulation (50) in particular but also malnutrition (51, 52) and genetic influences (53). On the environmental side especially culture (54) and family factors (e.g., involvement, communication, supportive Relationships, enable relationships (55, 56), psychopathology (57), maltreatment (58), single-parent and socioeconomic status (59).

Like social skills, bonding is also influenced by a number of different factors [e.g., parental behavior, nutrition (60), maternal personality (61)]. As documented earlier, the short-term effects of a lack of bonding are largely negative, but the fact that it can have even a long-term to lifelong influence on a connection with social skills and thus, as we know with regard to the positive function of social skills [ranging from school readiness (62) to academic success (63) and its protective function against developmental psychopathology (64)] seems considerable. This is meaningful because some studies found positive effects of bonding for a short time, e.g., a few days after birth, but which were no longer present weeks later (65) and which may cast doubt on the effectiveness of bonding. Interestingly, correlations with juvenile deliquence can be found for both poor bonding (66) and social skills (27). According to Mak (67) caring mothers who are perceived as warm and understanding are a protective factor against deliquence. In addition, there is evidence that paternal violence, physical abuse and sexual abuse by the father increased adolescents' sexual aggression, whereas bonding to the mother decreased it (68).

The influence of neuropeptides (especially oxytocin (69–76) as one possible mediator of this association, also arginine vasopressin [AVP]), an altered GABAA inhibitory system and steroids (especially oestrogens) in the first social phase on later behavior and emotion regulation has been documented extensively (69–71, 77). Oxytocin arguably stimulates maternal feelings (78), influences maternal behavior (79–83), promotes bonding (84, 85) and at the same time is itself derived from early social interaction (1, 86). Several studies using intranasal oxytocin administration show significant improvements in social skills (87) in the case of rats administered to the central amygdala (88), children and adolescents with autism spectrum disorders (89), others in case of children and adolescents with autism spectrum disorders not (90, 91).

Another mediator of this association might be maternal psychopathology: it has been demonstrated, that psychopathology of the mother can have a negative impact on the quality of bonding with the child [e.g., postnatal anxiety (92), depression at 2 and 6 weeks as well as 4 months (93)]. The study by Galeshi et al. (94) also shows that anxiety, depression and unwanted pregnancy are influencing factors on mother-child bonding and suggests that early diagnosis and treatment of maternal anxiety and depression has a positive effect on bonding. Genetically similar depressive symptoms in children are in turn associated with deficient social skills and problems with peers (95). Conversely, a very high level of prosocial behavior, especially in the case of low social participation, can in contrast promote the development of emotional symptoms (96). Also, as data in our study are based on maternal report only, maternal depression could have lead to maternal perceptive distortions and as such, a more negative perception of bonding and social competences. Furthermore, Bonding has been shown to be related to maternal Psychopathology (93). However, in our psychosocially well-adjusted community sample mothers showed remarkably low levels in terms of psychopathology according to SCL-90-R-cut-off, therefore a distinct relation between bonding and social competences can still be assumed, although this association needs to be elucidated in further studies e.g., on mothers with a significant degree of psychopathology.

The majority of definitions of bonding emphasize the emotional component (3). In the case of babies, therefore, not only the physical satisfaction of needs should be taken into account, more importance should be attached to social interaction. Babies who lack social interaction may do not gain enough weight, become indifferent, listless, withdrawn and develop psychopathology (e.g., depression) (97).

All mothers in our sample were married to the childs' father and had rather good bonding qualities. Children were healthy full term births. It remains to elucidate whether the association demonstrated in this study can also be found in higher risk samples.

There is a lack of studies investigating whether interventions to promote mother-child bonding in early infancy improve the social skills of preschool-aged children.

### Strengths and Limitations of the Study

Our results indicate the existence of a -theoretically assumed- relationship between bonding and social skills even in a psychosocially very well-adjusted and healthy community sample. Even in this sample, it is evident that successful bonding seems to increase the social competence of children 5.5 years later. Also, with regard to the multitude of further factors that influence our social competences, the extent of the correlation seems considerable. One major limitation is the fact, that bonding as well as social competences were assessed in maternal judgment. Therefore, a general distortion of maternal perception cannot be excluded. Paternal variables were not assessed. Due to the specificity of the present sample, additional studies (including a clinical sample) are necessary to verify the causality of the relationship. The inclusion of other variables (e.g., ability to regulate emotions, anxiety, temperament, environmental influences, biological factors) would be meaningful in future long-term studies. With regard to the instruments, in order to be able to classify the distribution of the sample with the SOCOMP, provisional cut-off values were assigned, which may influence the results.

## Data Availability Statement

The raw data supporting the conclusions of this article will be made available by the authors, without undue reservation.

## Ethics Statement

The studies involving human participants were reviewed and approved by University of Heidelberg, Medical School Ethics Committee. Written informed consent to participate in this study was provided by the participants' legal guardian/next of kin.

## Author Contributions

EM recruited participants and collected the data. JJ performed the statistical analysis and wrote the first draft of the manuscript. All authors had full access to all the data in the study and take responsibility for the integrity of the data and the accuracy of the data analysis. Furthermore, all authors read, gave feedback, and approved the submitted version.

## Conflict of Interest

The authors declare that the research was conducted in the absence of any commercial or financial relationships that could be construed as a potential conflict of interest.

## Publisher's Note

All claims expressed in this article are solely those of the authors and do not necessarily represent those of their affiliated organizations, or those of the publisher, the editors and the reviewers. Any product that may be evaluated in this article, or claim that may be made by its manufacturer, is not guaranteed or endorsed by the publisher.
